# Prenatal and Postnatal Exposure to Persistent Organic Pollutants and Infant Growth: A Pooled Analysis of Seven European Birth Cohorts

**DOI:** 10.1289/ehp.1308005

**Published:** 2015-03-06

**Authors:** Nina Iszatt, Hein Stigum, Marc-André Verner, Richard A. White, Eva Govarts, Lubica Palkovicova Murinova, Greet Schoeters, Tomas Trnovec, Juliette Legler, Fabienne Pelé, Jérémie Botton, Cécile Chevrier, Jürgen Wittsiepe, Ulrich Ranft, Stéphanie Vandentorren, Monika Kasper-Sonnenberg, Claudia Klümper, Nynke Weisglas-Kuperus, Anuschka Polder, Merete Eggesbø, OBELIX

**Affiliations:** 1 Division of Epidemiology, Norwegian Institute of Public Health, Oslo, Norway; 2 Department of Occupational and Environmental Health, School of Public Health, Université de Montréal, Montreal, Quebec, Canada; 3 Université de Montréal Public Health Research Institute (IRSPUM), Université de Montréal, Montreal, Quebec, Canada; 4 Environmental Risk and Health, Flemish Institute for Technological Research (VITO), Mol, Belgium; 5 Department of Environmental Medicine, Faculty of Public Health, Slovak Medical University, Bratislava, Slovakia; 6 Department of Environmental Medicine, University of Southern Denmark, Odense, Denmark; 7 Department of Biomedical Sciences, University of Antwerp, Antwerp, Belgium; 8 Institute for Environmental Studies (IVM), VU University Amsterdam, Amsterdam, the Netherlands; 9 Inserm, UMR 1085, IRSET, Rennes 1 University, Rennes, France; 10 Service d’Epidémiologie et de Santé Publique, Centre Hospitalier Universitaire de Rennes, Rennes, France; 11 Team “Early Origin of the Child’s Health and Development” (ORCHAD), INSERM, UMR1153 Epidemiology and Biostatistics Sorbonne Paris Cité Center (CRESS), Paris Descartes University, Paris, France; 12 Faculty of Pharmacy, University Paris Sud, Châtenay-Malabry, France; 13 Department of Hygiene, Social and Environmental Medicine, Ruhr-University Bochum, Bochum, Germany; 14 IUF–Leibniz Research Institute for Environmental Medicine, Düsseldorf, Germany; 15 Environment and Health Department, Institute for Public Health Surveillance, Saint-Maurice Cedex, France; 16 Division of Neonatology, Department of Pediatrics, Erasmus MC – Sophia Children’s Hospital, Rotterdam, the Netherlands; 17 Department of Food Safety and Infection Biology, Norwegian University of Life Sciences, Ås, Norway

## Abstract

**Background:**

Infant exposure to persistent organic pollutants (POPs) may contribute to obesity. However, many studies so far have been small, focused on transplacental exposure, used an inappropriate measure to assess postnatal exposure through breastfeeding if any, or did not discern between prenatal and postnatal effects.

**Objectives:**

We investigated prenatal and postnatal exposure to POPs and infant growth (a predictor of obesity).

**Methods:**

We pooled data from seven European birth cohorts with biomarker concentrations of polychlorinated biphenyl 153 (PCB-153) (*n* = 2,487), and *p,p*´-dichlorodiphenyldichloroethylene (*p,p*´-DDE) (*n* = 1,864), estimating prenatal and postnatal POPs exposure using a validated pharmacokinetic model. Growth was change in weight-for-age *z*-score between birth and 24 months. Per compound, multilevel models were fitted with either POPs total exposure from conception to 24 months or prenatal or postnatal exposure.

**Results:**

We found a significant increase in growth associated with *p,p*´*-*DDE, seemingly due to prenatal exposure (per interquartile increase in exposure, adjusted β = 0.12; 95% CI: 0.03, 0.22). Due to heterogeneity across cohorts, this estimate cannot be considered precise, but does indicate that an association with infant growth is present on average. In contrast, a significant decrease in growth was associated with postnatal PCB-153 exposure (β = –0.10; 95% CI: –0.19, –0.01).

**Conclusion:**

To our knowledge, this is the largest study to date of POPs exposure and infant growth, and it contains state-of-the-art exposure modeling. Prenatal *p,p*´*-*DDE was associated with increased infant growth, and postnatal PCB-153 with decreased growth at European exposure levels.

**Citation:**

Iszatt N, Stigum H, Verner MA, White RA, Govarts E, Palkovicova Murinova L, Schoeters G, Trnovec T, Legler J, Pelé F, Botton J, Chevrier C, Wittsiepe J, Ranft U, Vandentorren S, Kasper-Sonnenberg M, Klümper C, Weisglas-Kuperus N, Polder A, Eggesbø M, OBELIX. 2015. Prenatal and postnatal exposure to persistent organic pollutants and infant growth: a pooled analysis of seven European birth cohorts. Environ Health Perspect 123:730–736; http://dx.doi.org/10.1289/ehp.1308005

## Introduction

Rapid weight gain during the first few months of life is a predictor for later obesity (Monteiro and Victora 2005). Perinatal exposure to chemicals may contribute to obesity by affecting endocrine and neuronal pathways (La Merrill and Birnbaum 2011). Polychlorinated biphenyls (PCBs), *p,p´*-dichlorodiphenyltrichloroethane (*p,p´*-DDT) and metabolite *p,p´*-dichlorodiphenyldichloroethylene (*p,p´*-DDE) were used in agriculture and industry until global efforts in the 1990s to eliminate them (Stockholm Convention 2001). Substantial exposure is still observed due to the persistency of these lipophilic compounds (Longnecker et al. 2003). PCB-153 has a biological half-life of about 14 years (Ritter et al. 2011), and *p,p´*-DDE about 13 years (Wolff et al. 2000).

Epidemiological studies on perinatal POPs exposure, growth, and obesity have focused on transplacental exposure, with inconsistent results for PCB and a predominantly positive association for *p,p´-*DDE (reviewed by Cupul-Uicab et al. 2013). However, substantial exposure occurs postnatally through breastfeeding, research on which is limited (Grandjean et al. 2003; Jacobson et al. 1990; Pan et al. 2010; Patandin et al. 1998; Rogan et al. 1987). Most studies were limited by sample size and had no or incomplete postnatal exposure [assessed using the product of persistent organic pollutants (POPs) concentration and breastfeeding duration]. Postnatal exposure needs to be considered but is challenging because multiple factors such as child growth and maternal weight gain also influence children’s internal concentrations.

In the largest study to date on POPs and growth, we pooled data from seven European birth cohorts to investigate the association between prenatal and postnatal POPs exposure and infant growth from birth to 24 months in singleton term children, and used a recently developed pharmacokinetic model (Verner et al. 2013) to improve postnatal exposure assessment.

## Methods

*Description of cohorts*. Previously, Govarts et al. (2012) identified 14 cohorts of mother–child pairs with POPs measures from the Environmental Health Risks in European Birth Cohorts’ (ENRIECO) inventory (http://www.enrieco.org/). Eleven of these cohorts had relevant weight data and were invited to participate. Four [FAROES2, FAROES3, INMA (INfancia y Medio Ambiente; Environment and Childhood), Rhea] did not participate for reasons unrelated to this study hypothesis. Seven cohorts (*n* = 2,487) had PCB-153, and 5 cohorts (*n* = 1,864) had *p,p´-*DDE biomarkers. Concentrations were measured in cord serum/plasma in FLEHS I (Flanders Environment & Health Survey) (Koppen et al. 2009), GRD (Groningen–Rotterdam–Düsseldorf) (Huisman et al. 1995, Walkowiak et al. 2001), Michalovce (Hertz-Picciotto et al. 2003), and PELAGIE (Perturbateurs endocriniens: Étude Longitudinale sur les Anomalies de la Grossesse, l’Infertilité et l’Enfance) (Chevrier et al. 2013); in breast milk in ELFE (Growing up in France) (Vandentorren et al. 2009) and HUMIS (Norwegian Human Milk Study) (Eggesbø et al. 2009); and in maternal blood during pregnancy in Duisburg (Wilhelm et al. 2008, Wittsiepe et al. 2008). Table 1 lists population characteristics, and Supplemental Material, Table S1, contains cohorts’ descriptions and references. Each study was approved by national ethical committees. Mothers provided written informed consent prior to participation.

**Table 1:** Characteristics of the cohorts [median (range) or *n* (%)].

Characteristic	Duisburg(Germany,2000–2002)(*n* = 222)	ELFE (France,2007)(*n* = 35)	FLEHS I(Belgium,2002–2004)(*n* = 134)	GRD(Germany, Netherlands,1990–1995)(*n* = 588)	HUMIS(Norway,2002–2006) (*n* = 399)	Michalovce(Slovakia,2002–2004)(*n* = 938)	PELAGIE(France,2002–2006)(*n* = 171)
Change in weight-for-age *z*-score 0–24 months	–0.13 (–2.74–4.83)	0.06 (–2.38–2.21)	0.04 (–2.59–3.40)	–0.03 (–3.87–3.46)	–0.03 (–3.91–3.83)	–0.01 (–5.01–3.72)	0.03 (–2.85–3.63)
Missing (%)	0	0	0	0	1	3	0
Weight 24 m (kg)	13.56 (10.35–17.50)	12.00 (9.93–13.47)	12.53 (10.01–15.87)	13.21 (9.89–18.54)	12.19 (8.36–18.49)	13.66 (10.08–20.26)	12.39 (8.86–16.78)
Missing (%)	0	0	0	0	1	2	0
Height 24 m (cm)	87.4 (76.6–95.8)	87.6 (82.9–104.7)	88.0 (77.7–94.4)	88.6 (80.5–98.1)	86.3 (71.2–108.3)	86.2 (74.9–96.3)	87.5 (78.8––96.3)
Missing (%)	0	0	0	0	1	2	0
Birth weight (g)	3.460 (1.960–4.925)	3.340 (2.800–4.110)	3.383 (2.600–4.530)	3.500 (2.140–5.000)	3.680 (2.030–5.100)	3.370 (2.060–5.060)	3.370 (2.320–4.760)
Missing (%)	0	0	0	0	0	3	0
Birth length (cm)	52 (42–61)	49 (47–52)	50.7 (48.2–53.1)	52.5 (44–60)	51 (42–55)	50 (40–57)	50 (43–55)
Missing (%)	0	0	0	0	10	22	0
Gestational age (weeks)	40 (37–42)	40 (38–41)	40 (37–41)	40 (37–43)	40 (37–44)	40 (37–43)	40 (37–42)
Missing (%)	1	0	0	0	5	10	0
Sex
Male	108 (48.7)	18 (51.4)	72 (53.7)	322 (54.8)	203 (51.1)	477 (51.0)	92 (53.8)
Female	114 (51.4)	17 (48.6)	62 (46.3)	266 (45.2)	195 (49.0)	459 (49.0)	79 (46.2)
Missing (%)	0	0	0	0	1	2	0
Maternal age (years)	31.9 (19.3–42.6)	32.9 (24.3–41.3)	31.1 (20.3–41.1)	29 (18–40)	29 (16–42)	25.7 (17.9–45)	31.1 (20.1–45)
Missing (%)	0	0	0	0	0	11	0
Maternal prepregnancy BMI	23.0 (14.9–51.4)	21.5 (18.1–27.7)	22.4 (16.9–37.4)	22.1 (15.0–48.3)	23.3 (16.6–43.8)	21.2 (14.5–40.7)	21.9 (17.3–37.6)
Missing (%)	0	1	1	1	6	44	0
Maternal prepregnancy weight (kg)	65 (42–140)	58 (47–78)	62 (45–112)	64 (44–133)	66 (43–120)	58 (38–115)	58 (46–105)
Missing (%)	0	0	1	1	4	44	0
Maternal gestational weight gain (kg)	NA	NA	15.0 (1.0–30.0)	NA	14.0 (–3.0–31.0)	14.0 (1.0–35.0)	13.0 (5.0–31.0)
Missing (%)	222	35	60	588	8	250	0
Maternal height (cm)	168 (151–183)	163 (148–173)	168 (150–183)	170 (150–193)	168 (149–199)	165 (133–186)	165 (150–190)
Missing (%)	0	1	1	0	3	0	0
Parity
0	0 (0.0)	12 (34.3)	84 (62.7)	294 (50.0)	154 (38.6)	391 (41.7)	68 (39.8)
≥ 1	222 (100.0)	23 (65.7)	50 (37.3)	294 (50.0)	245 (61.4)	547 (58.3)	103 (60.2)
Missing (%)	0	0	0	0	0	1	0
Education
Low	50 (22.5)	1 (2.9)	5 (3.7)	73 (12.4)	53 (13.4)	177 (19.0)	29 (17.0)
Medium	83 (37.4)	8 (22.9)	96 (73.3)	184 (31.4)	253 (64.1)	687 (73.7)	26 (15.2)
High	89 (40.1)	26 (74.3)	30 (22.9)	330 (56.2)	89 (22.5)	68 (7.3)	116 (67.84)
Missing (%)	0	0	3	1	4	6	0
Maternal smoking during pregnancy^*a*^
No	169 (76.1)	33 (100.0)	120 (90.2)	438 (74.5)	357 (89.5)	805 (85.8)	137 (80.6)
Yes	53 (23.9)	0	13 (9.8)	150 (25.5)	42 (10.5)	133 (14.2)	33 (19.4)
Missing (%)	0	2	1	0	0	0	1
Ethnicity
Caucasian	211 (95.1)	NA	NA	588 (100.0)	374 (98.2)	738 (80.7)	171 (100.0)
Inuit	0	NA	NA	0	2 (0.5)	0	0
Roma	0	NA	NA	0	1 (0.3)	177 (19.3)	0
Other	11 (5.0)	NA	NA	0	4 (1.1)	0	0
Missing (%)	0	35	134	0	17	23	0
Sample type
Maternal blood	216	0	0	0	0	0	0
Cord blood	0	0	130	267	0	880	168
Breast milk	0	35	0	321	399	0	0
Missing (%)	6	0	4	0	0	58	3
Sample collection time (days from birth)	–51 (–107–42)	51 (36–70)	0 (0–0)	CB: 0 (0–0) BM: 14 (14–14)	32 (2–158)	0 (0–0)	0 (0–0)
Missing (%)	6	0	4	0	18	58	3
Total breastfeeding (months)	6.9 (0.2–18)	5.0 (1.6–23)	3 (0–48)	2.3 (0–6)	12 (1–31.2)	5 (0–48)	3.1 (0–24.8)
Missing (%)	38	2	42	5	0	5	8
No breastfeeding	0 (0)	0 (0)	34 (25.4)	224 (38.1)	0 (0)	2 (0.2)	70 (40.9)
Missing (%)	38	2	42	5	0	5	8
Exclusive breastfeeding (months)	4.8 (0–9.9)	NA	0 (0–1)	NA	5 (0–10)	3 (0–12)	NA
Missing (%)	39	35	76	588	0	3	171
Abbreviations: BM, breast milk; CB, cord blood; ELFE, Etude Longitudinale Française depuis l’Enfance (French Longitudinal Study of Children); FLEHS I, Flemish Environment and Health Survey I; GRD, Groningen–Rotterdam–Düsseldorf; HUMIS, Human Milk Study; NA, not available; PELAGIE, Endocrine disruptors: longitudinal study on pathologies of pregnancy, infertility and childhood. Continuous measures described by median (minimum–maximum); categorical measures described by frequencies (%).^***a***^In PELAGIE, smoking status at inclusion was used as proxy for smoking during pregnancy.							

*Exposure assessment*. All cohorts provided lipid-adjusted and wet-weight concentrations, plus information on lipid measurement (see Supplemental Material, Table S2). We replaced POP concentrations below the limit of detection/quantification (LOD/LOQ) (Table 2; see also Supplemental Material, Table S2) with LOD/LOQ divided by the square root of two (Hornung and Reed 1990).

**Table 2:** Infant blood concentrations for PCB-153 and *p,p’*-DDE prenatal and postnatal exposure, estimated through pharmacokinetic modeling (ng/g lipid).

Study	*n*	Prenatal PCB-153	< LOD*n* (%)	Postnatal PCB-153	*n*	Prenatal *p,p’*-DDE	< LOD*n* (%)	Postnatal *p,p’*-DDE
		Mean ± SD		Median		Mean ± SD		Median	Mean ± SD	Median	Mean ± SD	Median
Duisburg^*a*^	215	63.6 ± 45.9	56.7	0^*d*^	126.1 ± 98.8	108.7	215	141.4 ± 205.1	95.2	0^*d*^	255.3 ± 287.6	178.1
ELFE^*b*^	35	92.6 ± 41.9	83.3	0	301.1 ± 133.0	268.1	0	NA	NA	NA	NA	NA
FLEHS I^*c*^	129	54.0 ± 38.4	41.3	6 (4.5)^*d*^	66.5 ± 69.6	47.1	130	214.7 ± 244.5	145.6	0^*d*^	272.6 ± 412.9	150.6
GRD^*c*^	321	184.7 ± 72.9	176.7	0	280.4 ± 152.0	252.4	0	NA	NA	NA	NA	NA
HUMIS^*b*^	399	36.4 ± 17.1	33.1	0^*d*^	104.7 ± 52.2	96.8	399	63.4 ± 94.8	42.1	0^*d*^	177.3 ± 236.9	123.1
Michalovce^*c*^	880	164.4 ± 219.2	111.2	2 (0.2)	292.4 ± 425.4	175.3	880	540.5 ± 459.0	413.5	6 (0.7)	954.3 ± 1032.8	619.6
PELAGIE^*c*^	168	43.0 ± 31.5	32.1	0^*d*^	48.3 ± 55.9	26.2	168	73.5 ± 74.4	53.9	28 (16.4)^*d*^	75.7 ± 99.8	36.6
NA, not available. ^***a***^Prenatal and postnatal concentrations estimated from maternal blood concentrations. ^***b***^Prenatal and postnatal concentrations estimated from breast milk concentrations. ^***c***^Prenatal and postnatal concentrations estimated from cord blood concentrations. ^***d***^Provided LOQ instead of LOD.												

We estimated individual-specific cord blood concentration (prenatal exposure) and cumulative postnatal exposure using a validated pharmacokinetic model (Verner et al. 2013). Two compartments representing maternal and child lipids are connected through placental diffusion and excretion/intake of breast milk (see Supplemental Material, Figure S1A). To generate individual-specific profiles of child POPs concentrations (e.g., see Supplemental Material, Figure S1B), the pharmacokinetic model incorporated maternal age, prepregnancy weight, gestational age, sex, child’s weight at birth and several time points, and total breastfeeding duration (exclusive/partial). We used fixed values for gestational weight gain and postpartum maternal weight (Verner et al. 2013) because this information was not collected in some cohorts and therefore could not be imputed within cohort-specific multiple imputation models, in contrast with missing data for other covariates (see below). Most studies did not distinguish exclusive from partial breastfeeding; therefore, we used total breastfeeding duration. Breast milk consumption rate was based on exclusive/partial breastfeeding data from the general population (Arcus-Arth et al. 2005). Measured POP concentrations in maternal blood, cord blood, or breast milk (nanograms per gram lipids) were used to estimate individual-specific maternal daily dose and subsequently to simulate complete time course of child concentrations: Iterative model simulations adjusting the maternal daily dose provided matching simulated and measured blood or breast milk POP concentrations at the time of collection.

We abstracted the simulated cord blood concentration at delivery as the common prenatal exposure estimate across cohorts (vs. measures taken in different biological matrices sampled at different times). We calculated postnatal cumulative exposure 0–24 months as area under the curve (AUC) (see Supplemental Material, Figure S1B). We added prenatal (multiplied by gestational age) to postnatal to get total exposure from conception to 24 months. The three exposure metrics are expressed as average concentrations over their respective time periods (nanograms per gram lipids). Model simulations were performed using acsIX (Aegis Technologies Group Inc., Huntsville, AL, USA).

*Outcome variable*. Cohorts provided weight and height data with minimum two time points after birth to 3 years. Michalovce had data up to 4 years, Duisburg had data at 1.5 and 6.5 years. Weight and height data were measured for the study by nurses or doctors (GRD, Michalovce) or recorded during pediatric examinations in children’s health cards that were obtained by study staff (FLEHS I) or were parent-reported (Duisburg, ELFE, HUMIS, PELAGIE). We estimated weight at exactly 24 months using a cohort-specific, sex-specific, multilevel (mixed) linear model fitted with cubic polynomials and random effects for infant. We then created cohort-specific, sex-specific, weight-for-age *z*-scores at birth and 24 months. Intraclass correlations between predicted and observed values at 24 months (± 14 days) in infants with available data ranged from 0.84 (ELFE males) to ≥ 0.90 (ELFE females, FLEHS I, HUMIS, PELAGIE). GRD, Michalovce, and Duisburg had no children with observed data at 24 months. The difference between the child’s *z*-score at birth and 24 months (change in weight-for-age *z*-score) was analyzed as a continuous outcome.

*Statistical analysis*. We imputed missing data (outcome, exposure, covariate) by cohort, using multiple imputation by chained equations (ICE) (Rubin 1987; van Buuren 2007), and performed pharmacokinetic model simulations for each imputation set. The pharmacokinetic model used lipid-adjusted concentrations because they are more stable over time (Phillips et al. 1989). Some observations in all cohorts except ELFE and HUMIS were missing lipid-adjusted concentrations, and these were imputed in a cohort-specific model including wet-weight concentrations. We assessed correlations between exposures and covariates using Pearson’s correlation coefficients. We combined exposure, outcome, and covariate data from individual cohorts into a pooled data set to analyze as a single data set, using a multilevel (mixed) linear regression model to estimate associations between infant growth and separately, total, prenatal, and postnatal exposure. For each compound, we tested for heterogeneity by fitting a model with random intercepts and slopes by cohort. There was significant heterogeneity for all exposures (see Supplemental Material, Table S3). However, in the case of *p,p´*-DDE, which was available in only five cohorts, we had less power to fit a complex model [i.e., confidence intervals (CIs) were severely inflated; see Supplemental Material, Table S4]. Therefore, models were fitted with random intercepts for *p,p´-*DDE, and random intercepts and slopes for PCB-153. Models were fitted via maximum likelihood, using the STATA 12.0 “mi estimate” function to pool five imputation results. For prenatal and postnatal exposure, models were fitted first with either prenatal or postnatal concentrations in the model and then with both (prenatal and postnatal mutually adjusted). We checked for collinearity between prenatal and postnatal exposure with variance inflation factors (VIFs) greater than 5 to 10, suggesting a problem with collinearity (Kleinbaum et al. 2013).

We identified nine potential confounders and intermediate covariates *a priori* using directed acyclic graphs (DAGs) (see Supplemental Material, Figures S2A,B,C for total, prenatal, and postnatal exposure DAGs, respectively): maternal prepregnancy body mass index (BMI, continuous), maternal age (years, continuous), education (low, medium, high), smoking during pregnancy (yes/no), Roma ethnicity (yes/no), nulliparous (yes/no), gestational age (weeks, continuous), birth weight (kilograms, continuous), total breastfeeding (months, continuous), maternal gestational weight change (kilograms). Categories for primary and secondary education varied, so we combined categories to create relative low, medium, and high per cohort. ELFE and FLEHS I had no ethnicity information (important due to a large Roma population in Michalovce), so we assumed their ethnicity was not Roma. We made additional adjustment for maternal gestational weight change in FLEHS I, HUMIS, PELAGIE, and Michalovce. Similarly, we assessed the results’ sensitivity to maternal consumption of fatty fish (meals/week HUMIS, FLEHS I) and total fish (grams/week, PELAGIE, MICHALOVCE) by adjusting for these covariates.

We looked at the effect of removing each cohort in turn. We assessed assumptions of normality and linearity using informal diagnostic plots, and assessed the combination of high leverage and residuals in order to fit regression models with and without influential observations. Results are change in weight-for-age *z*-score from birth to 24 months for the interquartile range (IQR) of exposure.

## Results

Table 1 summarizes cohort characteristics. Duisburg infants were the slowest and ELFE infants the fastest growers. Total breastfeeding duration varied (2.3–12 months), as did number of infants with no breastfeeding: Duisburg, ELFE, and HUMIS had none; Michalovce, 0.2%; FLEHS I, 25.4%; GRD, 38.1%; and PELAGIE, 40.9%.

Table 2 shows estimated prenatal and postnatal infant blood POPs concentrations. For prenatal concentrations, PELAGIE was lowest and GRD highest for PCB-153, and HUMIS lowest and Michalovce highest for *p,p´*-DDE. PELAGIE had lowest postnatal exposures, whereas ELFE had highest PCB-153 and Michalovce highest *p,p´-*DDE. Measured biomarker concentrations were not substantially different from the estimated cord blood (prenatal) concentrations (see Supplemental Material, Table S5).

Prenatal and postnatal exposures were highly correlated (PCB-153 *r* = 0.71, *p,p´-*DDE *r* = 0.88; see Supplemental Material, Table S6), although this varied across the cohorts. Overall correlations between prenatal PCB-153/*p,p´-*DDE concentrations were moderate (*r* = 0.65), varying from *r* = 0.11 to *r* = 0.65 across cohorts (data not shown), whereas correlations between total breastfeeding and postnatal POPs exposure were lower (PCB-153 *r* = 0.43, *p,p´-*DDE *r* = 0.31) (see Supplemental Material, Table S6).

VIFs for prenatal and postnatal exposure in the same model varied across cohorts from low (i.e., FLEHS I, PELAGIE) to high (i.e., HUMIS, ELFE), and were < 5 for the pooled data set (see Supplemental Material, Table S7).

Table 3 shows the relation between total exposure from conception to 24 months and infant growth. Individual cohort analyses showed significant associations only for GRD (PCB-153) and Duisburg (*p,p´-*DDE). The pooled data showed nonsignificant associations between change in weight-for-age *z*-score and PCB-153 (β = –0.06; 95% CI: –0.15, 0.03 for an IQR increase of 152 ng/g lipid) and *p,p´-*DDE (β = 0.04; 95% CI: –0.001, 0.07 for an IQR increase of 515 ng/g lipid).

**Table 3:** Associations between total exposure from conception to 2 years to PCB-153 (152 ng/g) and *p,p’*-DDE (515 ng/g) and change in weight-for-age *z*-score.

Cohort	PCB-153	*p,p’*-DDE
	*n*	β (95% CI)	*n*	β (95% CI)
Duisburg	222	0.13 (–0.19, 0.46)	222	0.54 (0.22, 0.86)
ELFE	35	–0.19 (–0.74, 0.35)	0	NA
FLEHS I	134	0.06 (–0.50, 0.62)	134	0.05 (–0.22, 0.32)
GRD	588	–0.24 (–0.39, –0.09)	0	NA
HUMIS	399	–0.32 (–0.72, 0.08)	399	–0.26 (–0.56, 0.04)
Michalovce	938	0.01 (–0.02, 0.03)	938	0.02 (–0.02, 0.06)
PELAGIE	171	0.44 (–0.24, 1.12)	171	0.73 (–0.34, 1.81)
Pooled estimate (random)	2,487	–0.06 (–0.15, 0.03)	1,864	0.04 (–0.001, 0.07)
NA, not available. Results for both pooled sample and individual cohorts are per IQR increase for the pooled sample (ng/g lipid). Models were adjusted for birth weight, parity, gestational age, maternal smoking during pregnancy, maternal age at birth, maternal height and weight, Roma ethnicity and breastfeeding, and, for the pooled estimate, were fitted with random intercept (*p,p’*-DDE) and random intercept and slope (PCB-153) by cohort.				

**Figure 1:**
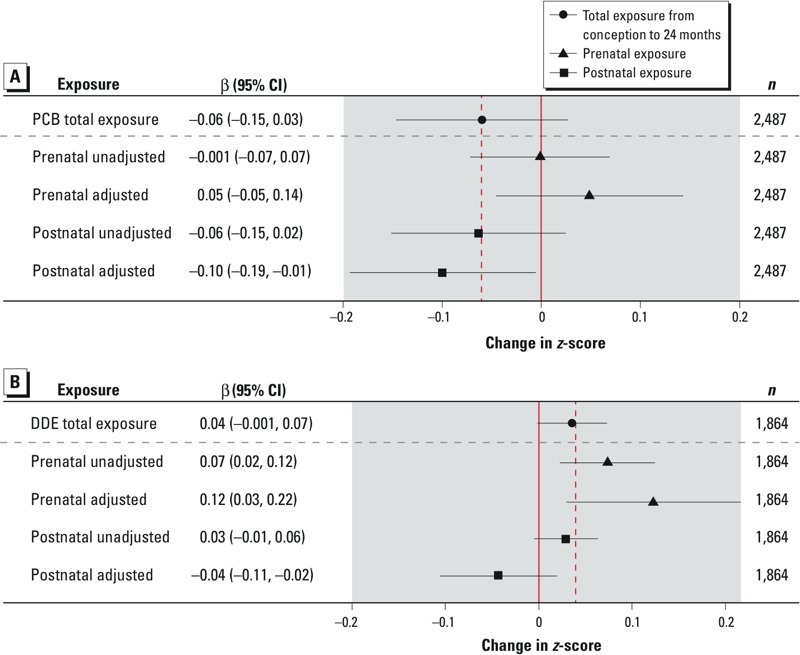
Associations between change in weight-for-age *z*-score and total exposure from conception to 2 years, prenatal exposure (unadjusted and adjusted for postnatal exposure) and postnatal exposure (unadjusted and adjusted for prenatal exposure) to (*A*) PCB-153 and (*B*) *p,p’*-DDE. Results are per IQR increase (ng/g lipid). PCB-153 IQRs: total exposure 152 ng/g, prenatal exposure 120 ng/g, postnatal exposure 183 ng/g. *p,p’*-DDE IQRs: total exposure 515 ng/g, prenatal exposure 388 ng/g, postnatal exposure 571 ng/g. Models were adjusted for birth weight, parity, gestational age, maternal smoking during pregnancy, maternal age at birth, maternal height and weight, Roma ethnicity, and breastfeeding, and were fitted with random slope (*p,p’*-DDE) and slope (PCB-153) by cohort. Prenatal unadjusted: prenatal exposure adjusted for covariates except postnatal. Prenatal adjusted: prenatal exposure adjusted for covariates including postnatal. Postnatal unadjusted: postnatal exposure adjusted for covariates except prenatal. Postnatal adjusted: postnatal exposure adjusted for covariates including prenatal.

Figure 1 shows the secondary analyses assessing which of prenatal and postnatal exposure is the more important contributor toward associations with total exposure. After adjustment for prenatal exposure, postnatal PCB-153 was associated with a significant decrease in change in weight-for-age *z*-score (β = –0.10; 95% CI: –0.19, –0.01 for an IQR increase of 183 ng/g lipid) (Figure 1A). Prenatal *p,p´-*DDE was associated with a significant increase in change in weight-for-age *z*-score (β = 0.12; 95% CI: 0.03, 0.22 for an increase of 388 ng/g lipid) after adjustment for postnatal exposure (Figure 1B).

In general, leaving out one cohort did not have a substantial influence on the prenatal and postnatal pooled estimates, with point estimates for the partial sample within the CI of the overall pooled result. However, for prenatal *p,p*´-DDE, removing Michalovce doubled the estimate (from 0.12; 95% CI: 0.03, 0.22 to 0.23; 95% CI: –0.09, 0.54) and made it nonsignificant (see Supplemental Material, Table S8).

Additional adjustment for fish consumption or maternal gestational weight change did not materially affect results (data not shown). Complete case and multiple imputation analyses gave essentially the same results, as did estimates of prenatal exposure from biomarker concentrations and pharmacokinetic modeled estimations (see Supplemental Material, Table S9). The normality and linearity assumptions of our models held (data not shown). There was no material difference in estimates from regression models fitted without influential observations (data not shown).

## Discussion

We found that prenatal *p,p´-*DDE exposure was significantly associated with increased infant growth, and postnatal PCB-153 exposure with decreased infant growth. Our estimates suggest that, on average, children with a 388-ng/g higher prenatal concentration of *p,p´-*DDE would weigh approximately 160 g more than other children at 24 months of age, whereas children with a 183-ng/g higher postnatal concentration of PCB-153 would weigh approximately 140 g less.

Prenatal PCB-153 concentrations were not significantly associated with infant growth. Because PCB-153 is a proxy biomarker for a number of PCB congeners of varying toxicity (Glynn et al. 2000), inconsistent results from previous studies (i.e., Blanck et al. 2002; Cupul-Uicab et al. 2010, 2013; Gladen et al. 2000; Hertz-Picciotto et al. 2005; Karmaus et al. 2009; Lamb et al. 2006; Mendez et al. 2011; Valvi et al. 2012, 2014; Verhulst et al. 2009; Warner et al. 2013) could be attributable to heterogeneity in the underlying PCB congeners mixture. Indeed, higher chlorinated PCBs have been associated with increased abdominal obesity in seniors, whereas the lower chlorinated PCBs showed an inverse relation (Lee et al. 2012).

The negative change in weight *z*-score associated with postnatal PCB-153 exposure is unlikely to be an an artifact of design (i.e., including weight in the pharmacokinetic model), because we did not find a significant association with postnatal *p,p´*-DDE exposure. Grandjean et al. (2003) also reported attenuated growth of breastfed children exposed to major PCB congeners (138, 153, and 180) at 18 months. Concentrations in that study were higher than in ours, whereas total breastfeeding duration was similar. However, four other studies reported no significant associations (Jacobson et al. 1990; Pan et al. 2010; Patandin et al. 1998; Rogan et al. 1987). Two studies investigated PCB concentration in formula-fed and breastfed babies (Jacobson et al. 1990; Patandin et al. 1998), whereas three estimated postnatal exposure simply as the product of PCB concentration and exclusive/total breastfeeding duration (Grandjean et al. 2003; Pan et al. 2010; Rogan et al. 1987). Exposure assessment that does not account for the dilution effect from weight increases in the growing child would lead to differential misclassification, overestimating exposure in the heavier children. Therefore, this would bias results upward, and, if the association between PCB-153 and growth is negative, could explain null findings reported in previous studies. The significant decline in birth weight of 150 g (95% CI: –250, –50) per 1-μg/L increase in PCB-153 reported by Govarts et al. (2012) indicates that PCB-153 may have a similar mode of action transplacentally and postnatally.

Prenatal *p,p´-*DDE exposure was significantly associated with increased change in weight-for-age *z*-score, a positive trend seen in four of the five cohorts. A change in *z*-score of 0.12 is modest, below the 0.67 cut point for rapid growth (Monteiro and Victora 2005). Conversely, postnatal *p,p´*-DDE exposure was not associated with infant growth, consistent with two previous studies (Pan et al. 2010; Rogan et al. 1987). Two limitations apply when interpreting this evidence. First, we found significant heterogeneity when pooling the cohorts in the *p,p´*-DDE analysis; however, fitting a more flexible model was not possible. Therefore, our estimate of the average effect does not account for the magnitude of variation among the cohorts. Second, although VIFs were < 5, variance doubled when prenatal and postnatal were mutually adjusted, suggesting collinearity. However, although our point estimate may be uncertain, the confidence intervals incorporate uncertainty generated by any collinearity (Kleinbaum et al. 2013). The more appropriate prenatal *p,p´*-DDE estimate probably lies between the unadjusted and adjusted one.

Our *p,p´*-DDE results are plausible. Most previous studies also found a positive association between *p,p´*-DDE and rapid growth and higher BMI in infancy (Mendez et al. 2011; Valvi et al. 2014; Verhulst et al. 2009), overweight and BMI around 7 years (Valvi et al. 2012; Warner et al. 2013), and weight-for-height at puberty in males (Gladen et al. 2000) and in adulthood for females (Karmaus et al. 2009). Studies with high concentrations, however, reported no significant associations (Cupul-Uicab et al. 2010, 2013; Garced et al. 2012; Gladen et al. 2004). This could be a chance finding or could suggest a mode of action that operates at lower doses, in line with the nonmonotonic relationship seen between endocrine-disrupting chemicals (EDCs) and hormones (Vandenberg et al. 2012). No studies to date reported a negative effect of *p,p´*-DDE on growth/BMI (Table S7 in Cupul-Uicab et al. 2013).

Our approach has strengths and limitations. We pooled data from seven European birth cohorts, examining associations between POPs and infant growth across larger samples of individuals with heterogeneous and distinct prenatal/postnatal exposure profiles. Compared with single-cohort studies, the pooled design controls better for unmeasured confounding (including from other compounds), because the underlying confounder structure varies across cohorts. Furthermore, it reduces or eliminates reporting bias by showing results for all eligible European cohorts.

We attempted to isolate prenatal from postnatal exposure; however, as discussed, the *p,p´*-DDE results suggest some collinearity. To ascertain a closer estimate of prenatal exposure, we would ideally restrict our analyses to babies who were not breastfed. Too few cohorts had non-breastfed babies (three for PCB-153, two for *p,p´-*DDE), precluding meaningful sensitivity analyses.

Our postnatal exposure modeling was more appropriate than the simple models used in previous studies, which incorporated only breastfeeding. The pharmacokinetic model generated exposure profiles based on cord blood, maternal blood, or breast milk levels and known determinants of children’s blood concentration. This model was validated in Michalovce and an Inuit cohort with repeated POPs measurements: Estimated concentrations from the pharmacokinetic model explained from 40% to 83% of *p,p´*-DDE and 51% to 81% of PCB-153 measured in children’s blood at 6 and 16 months (Verner et al. 2013). Estimations based on maternal blood were better than cord blood estimations, which were better than breast milk estimations. Repeated samples for model validation (e.g., cord/child blood concentrations) were unavailable in other cohorts in this study. It may be that the model predictability is lower in these cohorts, and model accuracy is lower in cohorts with estimations made from cord blood/breast milk. Despite these limitations, pharmacokinetic modeling presents a major improvement on metrics that do not account for important parameters (i.e., change in child weight, breast milk consumption, or lipid content). We did not have POPs dietary exposure information; however, breastfeeding is the main determinant of infant blood concentrations (Ayotte et al. 2003). Few cohorts distinguished exclusive from partial breastfeeding; therefore, we used total duration and descriptions of breast milk consumption in the general population. Furthermore, information on gestational weight gain and weight changes after pregnancy—influential in sensitivity analyses (Verner et al. 2013)—were not available in most cohorts. These factors may have decreased model precision and could lead to small differential misclassification, overestimating heavier children’s blood concentrations and resulting in a positive bias (in this case toward the null).

We added together the prenatal and postnatal exposure AUCs to assess total exposure from conception to 2 years, which has not been investigated in previous studies. Our prenatal AUC was calculated as the cord blood exposure estimate multiplied by gestational age. Although prenatal concentrations would be influenced by maternal weight gain over pregnancy, which would vary the volume of distribution, this information was not available in some of the cohorts. Our prenatal AUC is not expected to be more biased than using cord blood concentration as a proxy for prenatal exposure. Furthermore, adjustment for maternal gestational weight gain in cohorts where this was available did not affect our results (data not shown).

We used lipid-adjusted POP concentrations in our pharmacokinetic model, assuming an equilibrium across body lipids. Differential transport, or protection of the placenta or mammary gland due to molecular size, could require a conversion factor. However, considerable uncertainty is associated with conversion factors because of variability from factors other than differing measurement matrices (i.e., maternal body weight), which are not taken into account. Study-specific conversion factors are also difficult to apply to other studies with differing distributions of underlying co-factors. We therefore decided against applying conversion factors.

In addition, we were not able to test for exposure to combinations of these POPs and other EDCs, and may have missed important mixture effects.

We modeled weight at 24 months using a mixed model for growth with cubic polynomials, standardizing the children’s weights using cohort-specific data. Excessive infant growth between 0 and 24 months has previously been identified as a risk factor for obesity at a later age (Monteiro and Victora 2005). We did not test growth from birth to 6 months or 12 months because only four cohorts had appropriate measurements, and possibly missed a critical window for growth trajectory (Botton et al. 2008).

We did not have information on fatty fish intake in all cohorts; however, restricting analysis to the four cohorts where this was available revealed no material difference (data not shown).

Breastfeeding duration unadjusted for other covariates is associated with reduced growth in our study. Breastfeeding is an important contributor to postnatal POP exposure and relates to nutritional intake and other socioeconomic factors. However, additional adjustment for breastfeeding duration in the postnatal PCB-153 model had limited impact on the estimates (i.e., β = –0.12; 95% CI –0.21, –0.03 vs. β = –0.10; 95% CI –0.19, –0.01). Breastfeeding reduced the estimated increase in infant growth from prenatal *p,p´-*DDE by 14% (i.e., β = 0.14; 95% CI: 0.06, 0.22 vs. β = 0.12; 95% CI: 0.03, 0.22), possibly reflecting increased PCB-153 breastfeeding exposure.

Michalovce is a large cohort, accounting for 38% (PCB-153) and 50% (*p,p´*-DDE) of our total population. Although Michalovce drives the precision of the associations, these relations held after removing these children from the analyses. The postnatal PCB-153 and prenatal *p,p´*-DDE estimates became nonsignificant, as expected with a large sample size reduction, and the latter doubled in size.

## Conclusion

In a large and heterogeneous European population, we found an increase in infant growth associated with prenatal *p,p´-*DDE and a decrease associated with postnatal PCB-153 exposure. To understand the importance of POPs breastfeeding exposure on health, future investigations should assess both prenatal and postnatal exposure.

## Supplemental Material

(1.4 MB) PDF
