# Supplementary material for: Prenatal and Postnatal Exposure to Persistent Organic Pollutants and Infant Growth: A Pooled Analysis of Seven European Birth Cohorts
Source: Environ Health Perspect. 2015 Mar 6;123(7):730–6. doi: 10.1289/ehp.1308005 (PMC4492262; doi:10.1289/ehp.1308005)
Supplement: (1.4 MB) PDF [file ehp.1308005.s001.acco.pdf]

Environ Health Perspect
doi: 10.1289/ehp.1308005

Note to Readers: ***EHP*** **strives to ensure that all journal content is accessible to all readers. However, some figures and Supplemental Material published in** ***EHP*** **articles may not conform to 508 standards due to the complexity of the information being presented. If you need assistance accessing journal content, please contact ehp508@niehs.nih.gov. Our staff will work with you to assess and meet your accessibility needs within 3 working days.**

Supplemental Material

Prenatal and Postnatal Exposure to Persistent Organic Pollutants and Infant Growth: A Pooled Analysis of Seven European Birth Cohorts

Nina Iszatt, Hein Stigum, Marc-André Verner, Richard A. White, Eva Govarts, Lubica Palkovicova Murinova, Greet Schoeters, Tomas Trnovec, Juliette Legler, Fabienne Pelé, Jérémie Botton, Cécile Chevrier, Jürgen Wittsiepe, Ulrich Ranft, Stéphanie Vandentorren, Monika Kasper-Sonnenberg, Claudia Klümper, Nynke Weisglas-Kuperus, Anuschka Polder,[17] Merete Eggesbø, and OBELIX

Table of Contents

Table S1. Description of the birth cohorts with biological PCB-153 and p,p'-DDE exposure biomarkers included in the present study.*
Table S2. Chemical-analytical methods and detection/quantification limits of the birth cohorts.*
Figure S1. A) Conceptual representation of the pharmacokinetic model and B) examples of blood POP levels in mothers and infants.*
*Adapted from Verner et al. 2013. Reproduced with permission from Environmental Health Perspectives. AUC area under the curve. Simulations were carried out with a maternal daily dose of 10 ng/kg body weight/day. Model assumptions: exclusive maternal exposure through diet; complete gastrointestinal absorption; exclusive and homogenous distribution of POPs in

maternal and child lipids with unlimited transplacental diffusion (due to lipophilicity). POPs elimination (e.g., fecal excretion, metabolism) was based on published half-life values. Breast milk consumption rate was based on exclusive/partial breastfeeding data from the general population (Arcus-Arth et al. 2005).

Table S3. Description of heterogeneity between cohorts modelled with random slope (deviation from fixed effect).

Table S4. Comparison of pooled results from models fitted with random intercepts (Model 1) or additionally random slope for PCB-153 and *p,p'*-DDE (Model 2).

Figure S2A. Directed acyclic graph of the association between infant growth and total exposure.

Figure S2B. Directed acyclic graph of the association between infant growth and prenatal exposure.*

*Birth weight and gestational age are intermediate variables between prenatal POP exposure and infant growth. We are interested in the effect of exposures on infants' postnatal growth, not one that may be merely a continuation of prenatal growth mediated via birth weight. We included these variables in the model to close the pathway from prenatal exposure to infant growth via birth weight so that the model estimates only the direct association between prenatal exposure and postnatal growth.

Figure S2C. Directed acyclic graph of the association between infant growth and postnatal exposure.

Table S5. Biomarker concentrations and cord blood POPs concentrations estimated in the pharmacokinetic model (ng/g lipid).

Table S6. Pearson's correlation coefficients between prenatal concentrations, postnatal exposure and total breastfeeding duration.

Table S7. Variance inflation factors (VIFs) for prenatal and postnatal exposure in the same model.

Table S8. Comparison of results: leaving out one cohort at a time for PCB-153 and *p,p'*-DDE.

Table S9. Comparison of results for PCB-153 and *p,p'*-DDE: complete case dataset, multiple imputation dataset, and biomarker (instead of modelled) concentrations as "prenatal exposure."

References

Table S1. Description of the birth cohorts with biological PCB-153 and p,p'-DDE exposure biomarkers included in the present study.*

Cohort	Setting location	Time period	Enrollment method	Exclusion criteria	Participation rate	Age weight / height data collection	Exposure assessment[a] Biological matrix	Time of collection	N[b] PCB-153	*p,p'*-DDE	MI set[c]	Source
GRD	THE NETHERLANDS (Groningen-Rotterdam)	1990-1992	During prenatal consultations in late pregnancy by obstetricians or midwifes	Serious illness during pregnancy; Congenital anomalies; Parity>3; Caesarian section	70%	0, 18, 30, 42 months	Cord plasma	At birth	195	-	418	(Huisman et al. 1995)
	GERMANY (Düsseldorf)	1993-1995	At delivery from the obstetrical wards of 3 Düsseldorf hospitals by 3 medical students	Serious illness during pregnancy; Congenital anomalies; Parity>3; Caesarian section	70%	0, 18, 30, 42 months	Cord serum	At birth	126	-	170	(Walkowiak et al. 2001)
DUISBURG	GERMANY (Duisburg)	2000-2002	Self-selected pregnant women within a predefined area mainly in Duisburg South	Twins	Unknown	0, 1.5, 6, 8, 9-10 years	Maternal blood	32nd week of pregnancy	215	215	222	(Wilhelm et al. 2008; Wittsiepe et al. 2008)
FLEHS I	BELGIUM (Flanders)	2002-2004	At delivery in maternities of 8 districts covering 20% of Flanders' area	Complications in delivery; Living less than 5 years in the area; Not Dutch reading	98%	0, 12, 18, 24, 30, 36 months	Cord plasma	At birth	129	130	130	(Koppen et al. 2009)
Michalovce	SLOVAKIA	2002-2004	At delivery in maternities of 2 districts, 1 with high PCB contamination (Michalovce), and another upwind and upstream of chemical facility with lower contamination (Svidnik).	Mothers with major illness; Severe congenital anomalies; Maternal age<18y; Living less than 5 years in the area; Parity>4	60%	0, 6, 18, 48 months	Cord serum	At birth	880	880	938	(Hertz-Picciotto et al. 2003)
HUMIS	NORWAY	2002-2006	Two-4 weeks after birth during the routine health visit at home	Non-fluent in Norwegian	64%	Average 7 measures collected across 0, 6, 12, 24 months	Breast milk	Mixture of multiple samplings (once a week during 2 months after birth)	399	399	399	(Eggesbo et al. 2009)
PELAGIE	FRANCE (Brittany)	2002-2006	During first prenatal visit by gynaecologists or obstetricians in the study area	Inclusion later than 19 weeks of pregnancy	80%	Average 12 measures collected between 0, 24 months	Cord serum	At birth	168	168	171	(Chevrier et al. 2013)
ELFE pilot	FRANCE	2007	At delivery in maternities	Maternal age<18y; Not French speaking	55%	0, 1, 9, 24, 36 months	Breast milk	One month after birth	35	-	35	(Vandentorren et al. 2009)

*Adapted from Govarts et al. 2012. Reproduced with permission from *Environmental Health Perspectives.* [a]Selection criteria for exposure assessment was availability of biological samples except for HUMIS (random selection in cohort, breastfeeding), PELAGIE (Stratified random selection of a subcohort among the live born cohort and availability of biological samples), ELFE (breastfeeding). [b]Number of live-born singleton term births with lipid-adjusted concentration levels. This differs from Govarts et al. (2012) as only those that were followed-up are included. [c]Number of live-born singleton term births with lipid-adjusted concentration levels after multiple imputations.

Table S2. Chemical-analytical methods and detection/quantification limits of the birth cohorts.*

Cohort	Matrix	Extraction (phase)	Gas chromatograph separation	Detector type	Method of lipid analysis	LOD PCB 153	LOD *p,p'*-DDE
DUISBURG	Maternal blood	Liquid-liquid	High resolution	HRMS	Gravimetric	5 ng/L**	5 ng/L**
ELFE pilot	Breast milk	Liquid-liquid	High resolution	MS	Gravimetric	0.885 ng/g lipid	NA
FLEHS I	Cord plasma	Solid	Low resolution	MS	Gravimetric/Enzymatic	20 ng/L**	20 ng/L**
GRD	Cord plasma/serum	Liquid-liquid	High resolution	ECD	ND	10 ng/L	NA
HUMIS	Breast milk	Liquid-liquid	High resolution	ECD	Gravimetric	~0.458 ng/g lipid**	~0.224 ng/g lipid**
Michalovce	Cord serum	Solid	High resolution	ECD	Enzymatic	3.4-15.49 ng/L	2.87-58.05 ng/L
PELAGIE	Cord serum	Solid	High resolution	MS	Enzymatic	10 ng/L**	50 ng/L**

*Adapted from Govarts et al. 2012. Reproduced with permission from *Environmental Health Perspectives.* **Provided LOQ instead of LOD.

NA not available; ECD electron capture detection; MS mass spectrometry; HRMS high resolution MS; LOD limit of detection; LOQ limit of quantification; ND not determined.

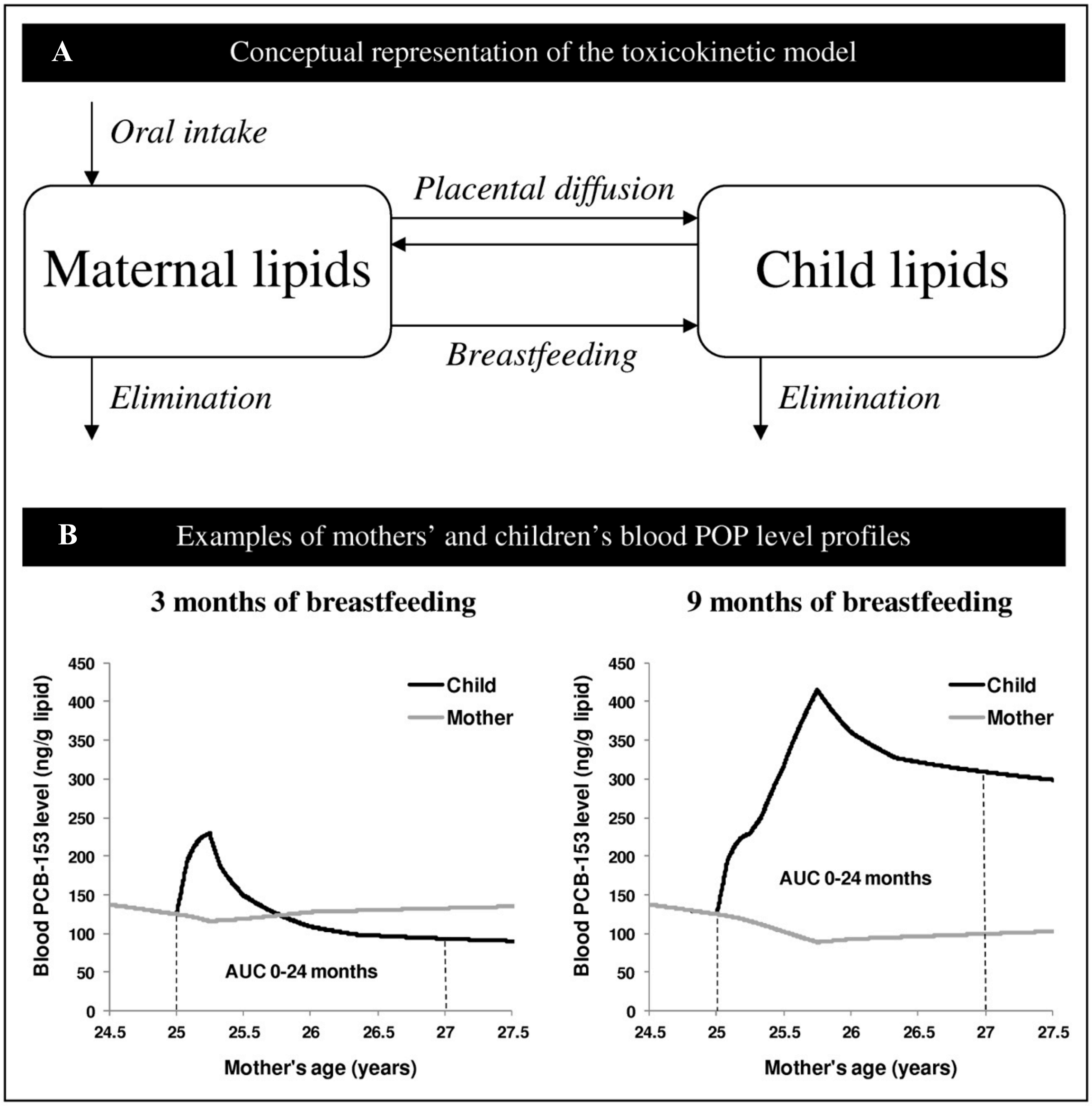

Figure S1. A) Conceptual representation of the pharmacokinetic model and B) examples of blood POP levels in mothers and infants.*

*Adapted from Verner et al. 2013. Reproduced with permission from Environmental Health Perspectives. AUC area under the curve. Simulations were carried out with a maternal daily dose of 10 ng/kg body weight/day. Model assumptions: exclusive maternal exposure through diet; complete gastrointestinal absorption; exclusive and homogenous distribution of POPs in maternal and child lipids with unlimited transplacental diffusion (due to lipophilicity). POPs elimination (e.g., fecal excretion, metabolism) was based on published half-life values. Breast

milk consumption rate was based on exclusive/partial breastfeeding data from the general population (Arcus-Arth et al. 2005).

Table S3. Description of heterogeneity between cohorts modelled with random slope (deviation from fixed effect).

	PCB-153		*p,p'*-DDE	
	**Prenatal**	**Postnatal**	**Prenatal**	**Postnatal**
*Standard deviation (95% CI) of random slope**				
	0.06 (0.01, 0.22)	0.05 (0.02, 0.16)	0.23 (0.09, 0.58)	0.19 (0.08, 0.46)
*Estimates of random slope deviation for each cohort***				
Duisburg	0.005	0.01	0.34	0.24
ELFE	-0.01	-0.01	NA	NA
FLEHS I	-0.002	0.004	-0.05	-0.03
GRD	-0.06	-0.04	NA	NA
HUMIS	0.003	-0.02	-0.27	-0.25
Michalovce	0.05	0.06	-0.02	-0.01
PELAGIE	0.01	0.01	-0.002	0.05

NA not available. Models adjusted for birth weight, parity, gestational age, maternal smoking during pregnancy, maternal age at birth, maternal height and weight, Roma ethnicity, total duration of breastfeeding, and postnatal exposure (for prenatal model) or prenatal exposure (for postnatal model), and fitted with random slope by cohort

*The random slope model estimates cohort-specific deviations from the overall fixed effect assuming a normal distribution, with the standard deviation listed above. If the standard deviation is significantly different from 0 then the cohorts significantly deviate from the overall fixed effect (i.e. significant heterogeneity). **The estimates of random slope deviation for each cohort are the realisations of the normal distribution describing how much each cohort deviates from the overall fixed effect.

Table S4. Comparison of pooled results from models fitted with random intercepts (Model 1) or additionally random slope for PCB-153 and *p,p'*-DDE (Model 2).

Compound	Exposure	N	Model 1<br>β (95 % CI)	Model 2<br>β (95 % CI)
PCB-153	Total exposure	2487	0.001 (-0.02, 0.03)	-0.06 (-0.15, 0.03)
	Prenatal	2487	0.07 (0.01, 0.13)	0.05 (-0.05, 0.14)
	Postnatal	2487	-0.05 (-0.10, 0.001)	-0.10 (-0.19, -0.01)
p,p'-DDE	Total exposure	1864	0.04 (-0.001, 0.07)	0.09 (-0.16, 0.35)
	Prenatal	1864	0.12 (0.03, 0.22)	0.11 (-0.15, 0.37)
	Postnatal	1864	-0.04 (-0.11, 0.02)	-0.002 (-0.23, 0.22)

Results per IQR increase (ng/g lipid). PCB-153 IQRs: total exposure 152 ng/g, prenatal exposure 120 ng/g, postnatal exposure 183 ng/g. p,p'-DDE IQRs: total exposure 515 ng/g, prenatal exposure 388 ng/g, postnatal exposure 571 ng/g.

Model 1 fitted with random intercept by cohort. Model 2 fitted with random intercept and random slope by cohort. Models adjusted for birth weight, parity, gestational age, maternal smoking during pregnancy, maternal age at birth, maternal height and weight, Roma ethnicity, total duration of breastfeeding, and postnatal exposure (for prenatal model) or prenatal exposure (for postnatal model), and fitted with random intercepts and slope by cohort.

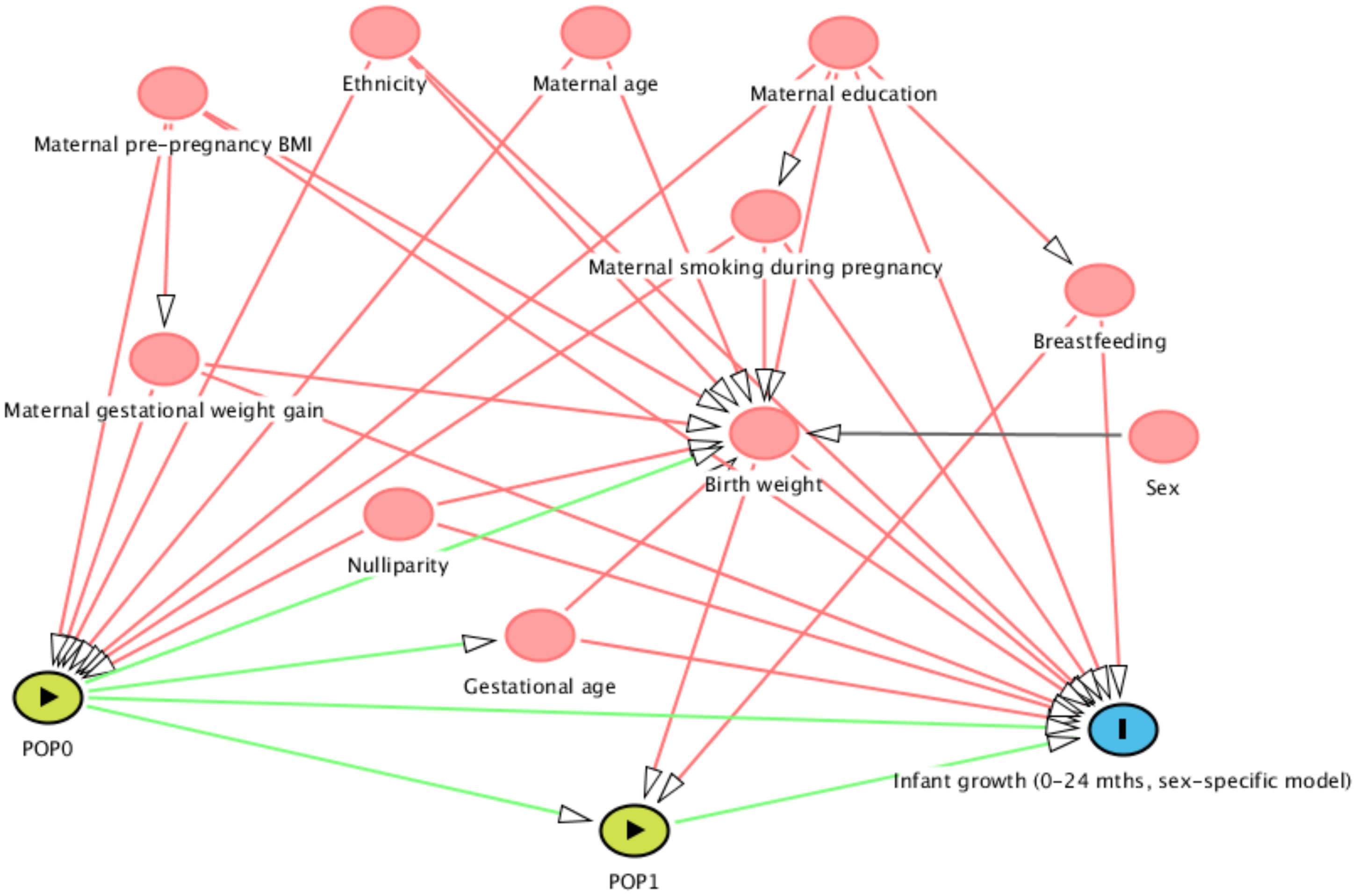

Figure S2A. Directed acyclic graph of the association between infant growth and total exposure.

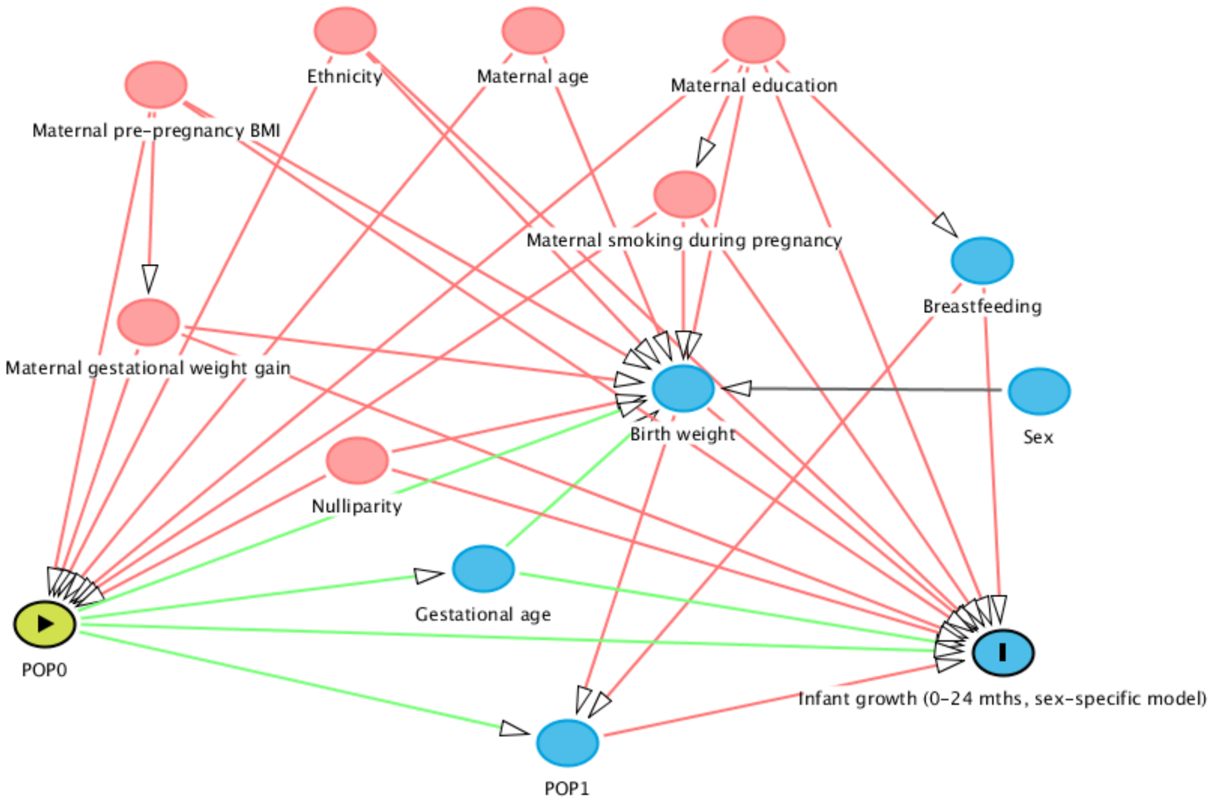

Figure S2B. Directed acyclic graph of the association between infant growth and prenatal exposure.*

*Birth weight and gestational age are intermediate variables between prenatal POP exposure and infant growth. We are interested in the effect of exposures on infants' postnatal growth, not one that may be merely a continuation of prenatal growth mediated via birth weight. We included these variables in the model to close the pathway from prenatal exposure to infant growth via birth weight so that the model estimates only the direct association between prenatal exposure and postnatal growth.

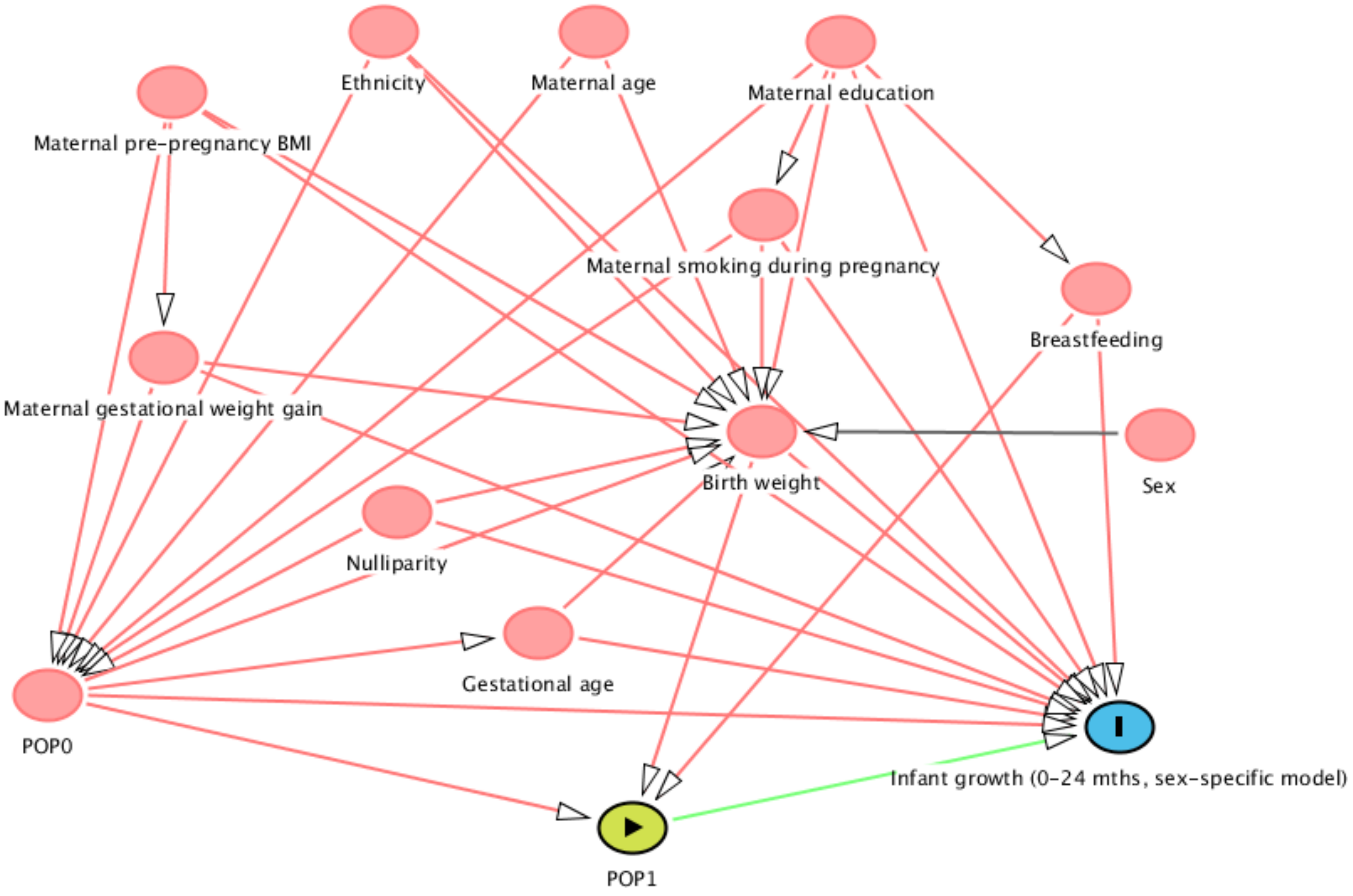

Figure S2C. Directed acyclic graph of the association between infant growth and postnatal exposure.

Table S5. Biomarker concentrations and cord blood POPs concentrations estimated in the pharmacokinetic model (ng/g lipid).

	PCB-153				*p,p'*-DDE			
**Cohort**	**N**	**Biomarker mean ±sd**	**Estimated mean ±sd**	**Difference (%)**	**N**	**Biomarker mean ±sd**	**Estimated mean ±sd**	**Difference (%)**
Duisburg[a]	215	65.7 ± 47.2	63.6 ±45.9	3.1 (1.6)	215	145.7 ± 210.2	141.4 ± 205.1	3.2 (1.6)
ELFE[b]	35	91.0 ± 40.9	92.6 ± 41.9	-1.7 (0.9)	0	-	-	-
FLEHS I[c]	129	54.0 ± 38.4	54.0 ± 38.4	0.0 (0.0)	130	214.8 ± 244.6	214.8 ± 244.6	0.0 (0.0)
GRD[c]	321	184.4 ± 72.7	184.4 ± 72.7	0.0 (0.0)	0	-	-	-
HUMIS[b]	399	36.0 ± 16.8	36.4 ± 17.1	-1.2 (1.1)	399	62.6 ± 93.5	63.4 ± 94.8	-1.2 (1.1)
Michalovce[c]	880	164.4 ± 219.3	164.4 ± 219.3	0.0 (0.0)	880	540.6 ± 459.1	540.6 ± 459.1	0.0 (0.0)
PELAGIE[c]	168	43.0 ± 31.5	43.0 ± 31.5	0.0 (0.0)	168	73.5 ± 74.4	73.5 ± 74.4	0.0 (0.0)

[a]Estimated from maternal blood concentration. [b]Estimated from breast milk concentration. [c]Cord blood concentrations.

Table S6. Pearson's correlation coefficients between prenatal concentrations, postnatal exposure and total breastfeeding duration.

**Compound**		**PCB-153**		**p,p'-DDE**	
	**Exposure**	**Prenatal**	**Postnatal**	**Prenatal**	**Postnatal**
PCB-153	Prenatal	1.00			
	Postnatal	0.71	1.00		
p,p'-DDE	Prenatal	0.65	0.49	1.00	
	Postnatal	0.57	0.73	0.88	1.00
Total breastfeeding duration		-0.15	0.43	-0.06	0.31

Table S7. Variance inflation factors (VIFs) for prenatal and postnatal exposure in the same model.

Compound	Exposure	Duisburg	ELFE	FLEHS	GRD	HUMIS	Michalovce	PELAGIE	Pooled
PCB-153	Prenatal	3.11	190.08	2.05	4.19	7.23	4.69	2.64	4.63
	Postnatal	4.33	185.85	3.5	10.81	7.81	5.25	3.86	4.92
p,p'-DDE	Prenatal	3.01	NA	3.33	NA	20.18	3.65	2.06	4.56
	Postnatal	3.45	NA	4.29	NA	21.57	4.38	2.92	4.65

NA not applicable.

Table S8. Comparison of results: leaving out one cohort at a time for PCB-153 and *p,p'*-DDE.

		PCB-153				*p,p'*-DDE		
**Analysis**	**N**	**Total exposure β (95% CI)**	**Prenatal exposure β (95% CI)**	**Postnatal Exposure β (95% CI)**	**N**	**Total exposure β (95% CI)**	**Prenatal exposure β (95% CI)**	**Postnatal Exposure β (95% CI)**
All cohorts	2487	-0.06 (-0.15, 0.03)	0.05 (-0.05, 0.14)	-0.10 (-0.19, -0.01)	1864	0.04 (-0.00, 0.07)	0.12 (0.03, 0.22)	-0.04 (-0.11, 0.02)
No Duisburg	2265	-0.07 (-0.18, 0.03)	0.03 (-0.09, 0.15)	-0.11 (-0.22, -0.00)	1642	0.02 (-0.02, 0.06)	0.11 (0.01, 0.20)	-0.04 (-0.11, 0.02)
No ELFE	2452	-0.06 (-0.15, 0.04)	0.05 (-0.05, 0.15)	-0.10 (-0.20, -0.00)	1864	0.04 (-0.00, 0.07)	0.12 (0.03, 0.22)	-0.04 (-0.11, 0.02)
No FLEHS I	2353	-0.07 (-0.16, 0.02)	0.04 (-0.06, 0.14)	-0.11 (-0.21, -0.01)	1730	0.04 (-0.00, 0.07)	0.12 (0.03, 0.21)	-0.04 (-0.10, 0.02)
No GRD	1899	0.01 (-0.02, 0.04)	0.06 (-0.01, 0.13)	-0.03 (-0.09, 0.02)	1864	0.04 (-0.00, 0.07)	0.12 (0.03, 0.22)	-0.04 (-0.11, 0.02)
No HUMIS	2088	-0.03 (-0.10, 0.03)	0.09 (0.03, 0.15)	-0.09 (-0.16, -0.01)	1465	0.05 (0.02, 0.09)	0.15 (0.06, 0.24)	-0.05 (-0.12, 0.01)
No Michalovce	1549	-0.09 (-0.21, 0.03)	0.04 (-0.15, 0.22)	-0.10 (-0.29, 0.08)	926	0.15 (-0.00, 0.30)	0.23 (-0.09, 0.54)	-0.02 (-0.29, 0.26)
No PELAGIE	2316	-0.08 (-0.17, 0.02)	0.03 (-0.07, 0.14)	-0.11 (-0.21, -0.01)	1693	0.03 (-0.00, 0.07)	0.12 (0.03, 0.22)	-0.05 (-0.11, 0.02)

Results per IQR increase for all cohorts (ng/g lipid). PCB-153 IQRs: total exposure 152 ng/g, prenatal exposure 120 ng/g, postnatal exposure 183 ng/g. p,p'-DDE IQRs: total exposure 515 ng/g, prenatal exposure 388 ng/g, postnatal exposure 571 ng/g.

Models adjusted for birth weight, parity, gestational age, maternal smoking during pregnancy, maternal age at birth, maternal pre-pregnancy BMI, Roma ethnicity, total duration of breastfeeding, and postnatal exposure (for prenatal model) or prenatal exposure (for postnatal model), and fitted with random intercepts (*p,p'*-DDE) and slope (PCB-153) by cohort

Table S9. Comparison of results for PCB-153 and *p,p'*-DDE: complete case dataset, multiple imputation dataset, and biomarker (instead of modelled) concentrations as "prenatal exposure."

		PCB-153				*p,p'*-DDE		
**Analysis**	**N**	**Total exposure β (95% CI**	**Prenatal exposure β (95% CI**	**Postnatal Exposure β (95% CI**	**N**	**Total exposure β (95% CI**	**Prenatal exposure β (95% CI**	**Postnatal Exposure β (95% CI**
Multiple imputation (MI)	2487	-0.06 (-0.15, 0.03)	0.05 (-0.05, 0.14)	-0.10 (-0.19, -0.01)	1864	0.04 (-0.001, 0.07)	0.12 (0.03, 0.22)	-0.04 (-0.11, 0.02)
Complete case	1971	0.01 (-0.01, 0.04)	0.09 (0.02, 0.15)	-0.12 (-0.23, -0.02)	1623	0.04 (-0.01, 0.08)	0.12 (0.03, 0.21)	-0.04 (-0.11, 0.02)
Biomarker concentration (MI)	2487	NA	0.05 (-0.05, 0.14)	NA	1864	NA	0.13 (0.03, 0.22)	NA

NA not available. Results per IQR increase for the imputed dataset (ng/g lipid). PCB-153 IQRs: total exposure 152 ng/g, prenatal exposure 120 ng/g, postnatal exposure 183 ng/g. p,p'-DDE IQRs: total exposure 515 ng/g, prenatal exposure 388 ng/g, postnatal exposure 571 ng/g.

Models adjusted for birth weight, parity, gestational age, maternal smoking during pregnancy, maternal age at birth, maternal pre-pregnancy BMI, Roma ethnicity, total duration of breastfeeding, and postnatal exposure (for prenatal model) or prenatal exposure (for postnatal model), and fitted with random intercepts (*p,p'*-DDE) and slope (PCB-153) by cohort.

References

Arcus-Arth A, Krowech G, Zeise L. 2005. Breast milk and lipid intake distributions for assessing cumulative exposure and risk. J Expo Anal Environ Epidemiol 15:357–365.

Chevrier C, Warembourg C, Gaudreau E, Monfort C, Blanc A Le, Guldner L, et al. 2013. Organochlorine pesticides, polychlorinated biphenyls, seafood consumption, and time-to-pregnancy. Epidemiol 24:251–260.

Eggesbø M, Stigum H, Longnecker M, Polder A, Aldrin M, Basso O, et al. 2009. Levels of hexachlorobenzene (HCB) in breast milk in relation to birth weight in a Norwegian cohort. Environ Res 109:559–566.

Govarts E, Nieuwenhuijsen M, Schoeters G, Ballester F, Bloemen K, Boer M de, et al. 2012. Prenatal exposure to polychlorinated biphenyls (PCB) and dichlorodiphenyldichloroethylene (DDE) and birth weight: A meta-analysis within 12 European Birth Cohorts. Environ Health Perspect 120:162–170.

Hertz-Picciotto I, Trnovec T, Kocan A, Charles M, Ciznar P, Langer P, et al. 2003. PCBs and early childhood development in Slovakia: study design and background. Fresenius Environ Bull 12:208–214.

Huisman M, Koopman-Esseboom C, Fidler V, Hadders-Algra M, Paauw C van der, Tuinstra L, et al. 1995. Perinatal exposure to polychlorinated biphenyls and dioxins and its effect on neonatal neurological development. Early Hum Dev 41:111–127.

Koppen G, Den H, Nelen V, Mieroop E Van De, Bruckers L, Bilau M, et al. 2009. Organochlorine and heavy metals in newborns: results from the Flemish Environment and Health Survey (FLEHS 2002–2006). Environ Int 35:1015–1022.

Vandentorren S, Bois C, Pirus C, Sarter H, Salines G, Leridon H. 2009. Rationales, design and recruitment for the Elfe longitudinal study. BMC Pediatr 9:58.

Verner M-A, Sonneborn D, Lancz K, Muckle G, Ayotte P, Dewailly É, et al. 2013. Toxicokinetic Modeling of Persistent Organic Pollutant Levels in Blood from Birth to 45 Months of Age in Longitudinal Birth Cohort Studies. Environ Health Perspect 121:131–137.

Walkowiak J, Wiener J, Fastabend A, Heinzow B, Kramer U, Schmidt E, et al. 2001. Environmental exposure to polychlorinated biphenyls and quality of the home environment: effects on psychodevelopment in early childhood. Lancet 358:1602–1607.

Wilhelm M, Wittsiepe J, Lemm F, Ranft U, Kramer U, Furst P, et al. 2008. The Duisburg birth cohort study: influence of the prenatal exposure to PCDD/Fs and dioxin-like PCBs on thyroid hormone status in newborns and neurodevelopment of infants until the age of 24 months. Mutat Res 659:83–92.

Wittsiepe J, Schrey P, Lemm F, Eberwein G, Wilhelm M. 2008. Polychlorinated dibenzo-p-dioxins/polychlorinated dibenzofurans (PCDD/Fs), polychlorinated biphenyls (PCBs), and organochlorine pesticides in human blood of pregnant women from Germany. J Toxicol Environ Health A 71:703–709.
